# The Diverse Forms of Lactose Intolerance and the Putative Linkage to Several Cancers

**DOI:** 10.3390/nu7095332

**Published:** 2015-08-28

**Authors:** Mahdi Amiri, Lena Diekmann, Maren von Köckritz-Blickwede, Hassan Y. Naim

**Affiliations:** 1 Department of Physiological Chemistry, University of Veterinary Medicine Hannover, Hannover D-30559, Germany; E-Mail: lena.diekmann@tiho-hannover.de; 2 The Research Center for Emerging Infections and Zoonosis (RIZ), University of Veterinary Medicine Hannover, Hannover D-30559, Germany; E-Mail: maren.von.koeckritz-blickwede@tiho-hannover.de

**Keywords:** lactase-phlorizin hydrolase, alactasia, adult type of hypolactasia, lactose intolerance, colorectal cancer, ovarian cancer, prostate cancer

## Abstract

Lactase-phlorizin hydrolase (LPH) is a membrane glycoprotein and the only β-galactosidase of the brush border membrane of the intestinal epithelium. Besides active transcription, expression of the active LPH requires different maturation steps of the polypeptide through the secretory pathway, including *N*- and *O*-glycosylation, dimerization and proteolytic cleavage steps. The inability to digest lactose due to insufficient lactase activity results in gastrointestinal symptoms known as lactose intolerance. In this review, we will concentrate on the structural and functional features of LPH protein and summarize the cellular and molecular mechanism required for its maturation and trafficking. Then, different types of lactose intolerance are discussed, and the molecular aspects of lactase persistence/non-persistence phenotypes are investigated. Finally, we will review the literature focusing on the lactase persistence/non-persistence populations as a comparative model in order to determine the protective or adverse effects of milk and dairy foods on the incidence of colorectal, ovarian and prostate cancers.

## 1. Introduction

The main sources of energy in our daily diet are carbohydrates, like starch, sucrose or lactose. The breakdown of starch molecules requires preliminary digestion by salivary and pancreatic amylases. These endoamylases only cleave α-1,4 glucosidic bonds. The final hydrolysis of di- and oligo-saccharides occurs by disaccharidases, which are located in the brush border membrane of enterocytes in the small intestine [1,2,3]. The three main intestinal disaccharidases are two α-glucosidases, (1) sucrase-isomaltase (SI) and (2) maltase-glucoamylase (MGA), and one β-glycosidase: lactase-phlorizin hydrolase (LPH). While SI is responsible for the cleavage of sucrose, isomaltose and 75%–80% of the hydrolysis of maltose, MGA mostly cleaves maltose. LPH accounts for 95% of lactase activity in the intestinal mucosa and additionally cleaves glycosylceramides [4]. The activities of SI and LPH are highest in the proximal intestine, whereas MGA has its highest activity in the ileum [5].

The hydrolysis of those ingested disaccharides, like lactose, is required for the uptake into the enterocytes. Only the constituent monosaccharides glucose, galactose and fructose are absorbable. The entry into the interior of the enterocytes occurs via carrier molecules. The uptake of glucose and galactose is mediated through the sodium/glucose cotransporter 1 (SGLT_1_) transporter, while fructose is carried by the GLUT transporter [6].

The defects of intestinal digestion of di- and oligo-saccharides (like congenital sucrase-isomaltase or lactase deficiency) or defects in the absorption of monosaccharides (like congenital glucose-galactose deficiency) lead to fermentative diarrhea, which constitutes the major symptoms associated with malabsorption. Undigested di- or oligo-saccharides or malabsorbed monosaccharides are osmotically-active molecules that lead to an increased flux of water into the gut. In the cecum, fermentation of the unabsorbed carbohydrate molecules occurs by colonic bacteria. This process, in turn, results in the production of H_2_, CO_2_ and fatty acids, which induce a further increase of the stool volume. The consequences of those different forms of malabsorption can range from mild to severe malnutrition and eventually death, depending on several factors, like the age of the patient and other exogenous factors.

In the first part of this review, we will discuss the structure, intracellular processing and functional features of the human lactase-phlorizin hydrolase protein, as well as the molecular genetics behind different types of LPH deficiencies. In the second part, a possible association of lactase persistent or lactase non-persistent phenotypes with the incidence of some common types of cancer is reviewed.

## 2. Molecular Aspects of Human Lactase-Phlorizin Hydrolase Protein

### 2.1. Structure and Function

LPH is a type I membrane glycoprotein, which is localized at the brush border membrane of enterocytes in the small intestine, where it fulfills its enzymatic digestive function [1,7]. While LPH, the only β-galactosidase in the intestinal lumen, cleaves specifically β-glucosidic linkages between monosaccharides, SI and MGA are α-glucosidases, which are responsible for the cleavage of α-glucosidic linkages [8,9,10]. The hydrolysis of oligo-and di-saccharides is required for the uptake across the brush border membrane to the cell interior and the further transport through the blood stream [11]. A possible digestive interaction has been shown to occur among the three disaccharidases in such a way that a decrease in the lactase activity level results in increased sucrase activity [12].

The lactase gene coding for human LPH is located on chromosome 2, including 49,340 base pairs with 17 exons [13,14]. After transcription and splicing, the mRNA contains 6274 bases and encodes for a polypeptide with 1927 amino acid residues [15]. LPH is a type I membrane glycoproteins that is synthesized as a single-chain precursor molecule in the endoplasmic reticulum (ER). The protein consists of an *N*-terminal extracellular domain, which comprises four homologues domains revealing 38%–55% identity to each other [15]. This high similarity among the four domains led to the assumption that LPH could have emerged from two subsequent duplications of a prokaryotic β-glycosidase gene [16]. LPH is anchored into the membrane via a membrane anchor built from 19 hydrophobic amino acids and ends with a 26 amino acid-long hydrophilic cytoplasmic domain [15]. The catalytic activities are located in domain III with the phlorizin-hydrolase activity at position Glu1273 and in domain IV with the lactase activity at position Glu1749 [10].

### 2.2. Biosynthesis and Trafficking

**Figure 1:**
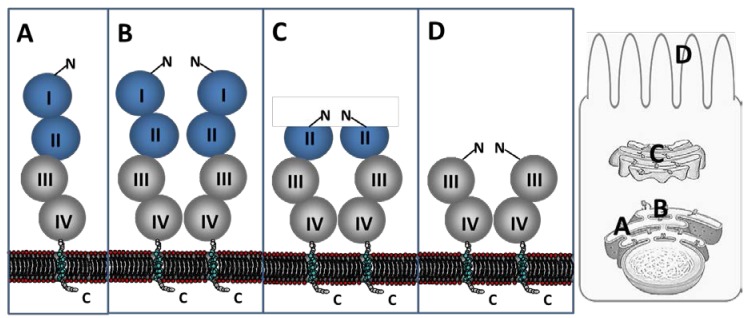
Maturation steps of lactase-phlorizin hydrolase (LPH) in the intestinal epithelial cells. (**A**) The protein is synthesized as a monomeric pro-LPH molecule by translocation in the endoplasmic reticulum (ER). LPH is a type I transmembrane glycoprotein, which consists of a luminal *C*-terminus, a membrane anchor and an ectodomain with four highly-conserved structural and functional domains and an extracellular *N*-terminus. In the ER, the polypeptide is *N*-glycosylated, which is required for the correct folding of the protein; (**B**) Prior to its exit from the ER, pro-LPH molecules form homodimers, which are required for the acquisition of transport-competence and enzymatic activity; (**C**) After transport to the Golgi apparatus, pro-LPH is cleaved in the *trans*-Golgi network, which leads to the removal of LPHα, leaving LPHβ_initial_. In addition, LPH is further *N*- and *O*-glycosylated, which is crucial for the correct folding, and subsequently, for the enzymatic activity of the protein; (**D**) After proper sorting of the protein to the apical membrane, LPHβ_initial_ is cleaved by pancreatic trypsin in the intestinal lumen to generate the mature form of the protein, called LPHβ_final_, consisting only of domains III and IV.

Different mammalian intestinal epithelial cells and different species were used to study the biosynthesis and the processing of LPH [1,17,18,19,20,21]. The primary structure of LPH is highly conserved among different mammalian species, so that human and rabbit LPH sequences have 82.6% identity and 90% similarity, and between human and rat LPH, these values are 77.6% and 86.7%, respectively (based on European Molecular Biology Open Software Suite (EMBOSS) Needle pairwise alignment). The synthesis of LPH starts in the ER, where it is post-translationally modified and then further transported along the secretory pathway. The protein is synthesized as a monomeric pro-LPH molecule with a molecular weight of 215 kDa (Figure 1A) [1]. The first co-translational modification in the ER is *N*-glycosylation, which plays an indispensable role in the correct folding of the protein [22]. The primary sequence of LPH consists of 15 *N*-glycosylation sites. Besides the correct folding of the protein, another requirement must be fulfilled in the ER before further transport of the protein. This is the formation of homodimers of two pro-LPH molecules, which is mediated by their transmembrane domains (Figure 1B) [23,24]. It is shown that dimerization is also required for the proper activity of LPH, before further trafficking to the Golgi apparatus [23,24].

In the Golgi apparatus, pro-LPH is complex *N*- and *O*-glycosylated, generating a 230-kDa pro-LPH protein [7,17]. It is known that *N*- and *O*-glycosylation are very important for correct folding, the transport, as well as the enzymatic activity of the protein [25,26]. Previous studies showed a four-fold increased activity of the *N*- and *O*-glycosylated form of LPH compared to the *N*-glycosylated form, indicating the importance of *O*-glycosylation for the enzymatic function of this protein [26]. The final post-translational processing of pro-LPH to the mature brush border form requires two proteolytic cleavage steps, which are not necessary for the activation and the transport competence of the protein [17,19]. One cleavage step occurs in the *trans*-Golgi network leading to the formation of LPHβ_initial_, where domains I and II, which are together known as the profragment LPHα, are cleaved off at position Arg734/Leu735 (Figure 1C). The profragment LPHα is known to function as an intramolecular chaperone, which is directly involved in the correct folding of LPHβ_initial_ [27]. LPHα is directly degraded after the intracellular cleavage step in the Golgi apparatus and is neither accessorily *N*-glycosylated, despite its five *N*-glycosylation sites, nor *O*-glycosylated [28]. It can be assumed that LPHα builds, due to its high content of hydrophobic amino acids, a compact, rigid and trypsin-resistant structure, which can mask the potential *N*-glycosylation sites directly after translation. There is no other individual role of LPHα known in the context of the whole protein function or its enzymatic activity [28,29]. LPHβ_initial_, which extends from Leu735 to Tyr1927, is further sorted to the apical membrane. The final cleavage occurs at the cell surface by pancreatic trypsin at position Arg868/Ala869 (Figure 1D). The remaining so-called LPHβ_final_ is a 160-kDa mature protein [1,21].

The homologous domain III of LPH is also known to function as an intramolecular chaperone. Elimination of this domain results in a misfolded protein, which is blocked in the ER. Domain III, which contains the phlorizin-hydrolase active site, is a fully-autonomous domain, which can be correctly sorted and transported, when expressed alone [30].

To achieve its physiological function, the hydrolysis of lactose, LPH must be transported to the luminal surface of the epithelial cells. Polarized sorting is often an event that is determined by specific sorting signals or by interacting with cellular components, such as membrane microdomains [31]. The transport to the apical surface of epithelial cells can be mediated via a glycophosphatidyl inositol (GPI) anchor, which associates in the trans-Golgi network with membrane microdomains enriched in glycosphingolipids and cholesterol [32,33]. The correct sorting of sucrase-isomaltase requires the association with lipid rafts, which is mediated by *O*-glycosylation of the protein [34,35]. The potential role of *N*-glycans to act as apical sorting signals was first described for the glycoprotein gp80, which is missorted after treatment with *N*-glycosylation inhibitors [36]. Interestingly, LPH does not associate with conventional lipid rafts, and it is known that *N*- and *O*-glycans have no effect on its apical sorting [22,32,37]. Transmembrane domain sequences are also known to be responsible for the apical sorting of different proteins, like, for example, the influenza virus neuraminidase [38]. The presence of the transmembrane region of LPH is one requirement for dimerization and, thus, also for sorting of LPH [39]. Neither the proteolytic cleavage step is implicated in the apical trafficking of LPH nor does the large profragment LPHα contain an apical sorting signal [19,40]. Recent studies strongly suggested that the sorting signals of LPH are located in domain IV, corresponding to the mature cleaved form, LPH_final_ [39,41]. It could be demonstrated that deletion of 236 amino acids of domain IV including the catalytic site has almost no influence on the sorting of LPH, while the further deletion of 87 amino acids leads to a missorting of the protein to the basolateral membrane. The region of the signal sequence was localized, but the exact amino acid sequence was not defined until now. It is known that apical membrane proteins are transported in distinct vesicular carriers, called SAVs (SI-associated vesicles) and LAVs (LPH-associated vesicles), when coming from the *trans*-Golgi network (TGN) [42,43]. Lectin proteins are postulated to serve as the sorting receptors that transiently cluster *N*-glycosylated proteins into apically-destined domains [44]. Galectin-3 is suggested to play an important role in the apical sorting as a sorting receptor by delivering non-raft-dependent glycoproteins to the lumen of LAVs in a carbohydrate-dependent manner [45].

## 3. Lactose Intolerance

Insufficient levels of lactase activity in the intestine lead to the inability to digest lactose from dairy products, especially milk. This type of malabsorption is called lactose intolerance and is classified into four different types. The first one is the primary lactase deficiency, also called adult hypolactasia, which appears in adulthood and is caused by the absence of a lactase persistent allele [46]. The second type is the congenital lactase deficiency, which is an autosomal recessive inherited disease that eliminates lactase activity already in infants [47,48]. The third type is the acquired or secondary lactase deficiency and is induced by an injury of the small intestine, e.g., from acute gastroenteritis, chemotherapy or infections with intestinal microbes [49]. Developmental lactase deficiency is the fourth form of lactose intolerance (LI), which occurs in preterm infants. In this type, birth occurs before lactase enzyme is optimally developed at term birth [49,50]. Compared to full-term infants, human fetuses have about 30% lactase activity at 26–34 weeks of gestation, which increases to 70% by 35–38 weeks [51,52]. Therefore, immature infants with less than 34 weeks of gestation suffer from maldigestion of lactose.

## 4. Adult Type of Hypolactasia

The prevalence of the primary adult type of hypolactasia (ATH) varies from less than 5% to almost 100% between different populations [53], but worldwide, an average of two-thirds of the adult population is affected [54]. The highest prevalence is detected in American Indians and Asians and up to 80% of Blacks, Arabs and Latinos reveal this disorder, while the lowest prevalence is detected in northwestern Europe [54]. Evolutionally, most mammals reach a peak of lactase activity directly after birth during lactation, when milk is the only nutrition. Usually, the lactase activity decreases to 5%–10% compared to the initial level between weaning and before adulthood, associated with changes in the composition of the diet. The persistence of lactase activity is most likely an event due to the “cultural-historical hypothesis”, proposing that diet conditions and natural selection in periods of diet stress are the reason for the permanent ability to digest lactose [55].

The persistence of lactase activity throughout life can be explained by a single autosomal dominant gene, allowing adults to accept high amounts of lactose in the diet [56,57]. Initial genotype/phenotype studies showed that two single nucleotide polymorphisms (SNPs) C/T_−13910_ and G/A_−22018_ substitutions in the *LCT* gene are associated with lactase persistence. Both nucleotide polymorphisms are localized upstream of the lactase gene and influence the *LCT* promotor [58,59]. While homozygotes with CC and GG stay non-persistent and show non-detectable lactase levels, the homozygotes with either TT or AA are persistent. Heterozygote carriers showed intermediate lactase levels with a wide range of varying symptoms [60]. This phenomenon is explained by *in vitro* experiments showing the strong binding of the transcription factor octamer-binding protein 1 (Oct-1) to the T_−13910_ variant, which can enhance the activity of the LCT gene promoter and increase the expression levels of LPH mRNA in the intestinal mucosa [46,61]. Meanwhile, the T_−13910_ variant seems to be associated with lactase persistent (LP) mostly in individuals of European origin. Later studies on populations from Africa and the Middle East have revealed different SNPs being associated with LP, including the G_−13907_, C_−13913_, G_−13915_, G_−14009_ and C_−14010_ variants [62,63,64,65,66]. Similar to the C_−13910_, C_−13913_ and G_−13915_ variants are located in the Oct-1 binding site (Positions −13922 to −13910), while the C_−14010_ variant is located more upstream in between the Oct-1 and hepatocyte nuclear factor 1-alpha (HNF1α) binding sites. All of these variants are suggested to activate the LCT promoter with a similar cis-acting effect via enhancing the Oct-1 factor binding and inducing chromatin changes in the vicinity of the LCT gene, resulting in the LP phenotype [61,62,64,66].

Besides the C_−13910_ genotype, studies have shown an association between C_−13779_ and G_−13806_ variants and the lactase non-persistent (LNP) phenotype in African and Indian populations [66,67]. However, there are many SNPs identified upstream of the LCT for which no functional characteristics have been reported yet: A_−13937_, A_−14107_, T_−14091_ and C_−14176_ detected in the South African population [68], T_−13801_, G_−14012_ and C_−14026_ identified in the Indian population [67] and T_−14011_ identified in Estonian and Indian individuals [69].

The symptoms of ATH patients range from mild symptoms to severe diarrhea and weight loss due to bacterial fermentation of undigested lactose and unabsorbed carbohydrates. Not all patients show these symptoms, although they suffer from the loss of lactase activity. In general, the intensity of the symptoms correlates with the consumed amount of lactose. Some individuals remain clinically unremarkable, because they can tolerate moderate amounts of lactose and lactose-containing nutrition [70]. Symptomatic lactose malabsorbers are presumed to have an additional susceptibility, e.g., for irritable bowel syndrome (IBS) [71,72,73]. It was shown that the bowel transit in patients with IBS is increased, which may also lead to the symptoms of lactose malabsorbers [74].

Nowadays, there are different established approaches available to diagnose lactose intolerance. The simplest and cheapest diagnostic tool is the lactose breath test, where the patient receives a defined amount of lactose. The undigested lactose is fermented by colonic bacteria and can be measured as hydrogen in the breath [75]. Another diagnostic method is the determination of lactase activity measured in small intestine tissue biopsy samples. Measurements below a lactase activity of 8 U/g or 0.7 U/g wet weight are defined to be associated with lactose intolerance [76]. This method is only reliable if the morphology of the mucosa and the enzymatic activities of the other disaccharidases, like MGA and SI, are in a normal range [77]. The latest method to detect lactose-intolerant patients is a genetic test of the C/T_−13910_ polymorphism [78]. The homozygous genotype CC determines hypolactasia. Nevertheless, it is very important to see the patient as a complex organism and to be sensitive to any additional influencing factors, which may lead to false-negative or false-positive results in the above-described test. The current recommended treatment of lactose-intolerant patients is the reduction of lactose consumption by diminishing lactose-containing products in the diet or to consume low-lactose or lactose-free food. It has been observed that ATH subjects drink less fresh milk compared to the persistent ones. This can affect energy and calcium levels, thereby eventually increasing the risk of osteoporosis [79,80]. The degree of the symptoms appearing is individually variable due to the consumed lactose and the personal lactase activity. People who remain symptomatic during complete lactose absence in the diet might be affected by other diseases, like IBS, celiac disease or bacterial overgrowth in the intestine [81].

## 5. Congenital Lactase Deficiency

Congenital lactase deficiency (CLD) is a severe and rare autosomal recessive disorder that leads to an elimination of lactase activity from birth onward. Most rationales for this disease are the appearance of truncated proteins as a result of frame shifts or missense mutations in the coding region of LPH [47,82,83]. There are a few cases described where a mutation led to a single amino acid substitution, which interfered with the function of LPH [84,85]. In a study with 32 Finish patients, five different mutations in the coding region of *LCT* were detected. One of them, called Fin_major_, was identified as a homozygous type in 84% of the patients. This mutation (Y1390X) leads to a truncated protein. Two further mutations (S1666fsX1722 and S218fsX224) result in a frame shift and a premature stop codon. The latest two mutations (Q268H and G1363S) generate an amino acid substitution [47,84]. The Fin_major_ mutation was analyzed by sequence comparison among 556 anonymous blood donors. The highest carrier frequency of 1:35 was found in a little town in Finland [47]. Noticeably, all of the other four mutations could not be detected in any regional subpopulation screening, except the G1363S mutation, which was found in another study in two siblings of Turkish origin in the homozygous state. This study also detected four other mutations, two in an Italian patient and two others in a Finish patient [85]. The G1363S-mutant was analyzed at the protein level, and it was demonstrated that this mutation leads to a misfolded protein that is blocked in the ER [84]. Recent studies have revealed two mutations in *LCT* in a Japanese infant with CLD, also resulting in a truncated protein [86]. Those two mutants, LPH-Y1473X and LPH-D1796fs, were also analyzed at the protein level and revealed that both proteins are misfolded and ultimately degraded in the ER. Interestingly, no interaction between the wild-type LPH monomer with one of the pathogenic LPH mutants was detectable, and the wild-type LPH generated normal homodimers that are enzymatically active, as well as transport competent [87]. Another new mutation (S1150fs) in *LCT* was recently found in a Turkish infant, resulting in a truncated protein [88]. Taking these findings under closer consideration, it may be hypothesized that the origin of the genetic background that lead to this severe and rare disease may be located in Finland.

Typical symptoms of a severe CLD start from a few days after birth by the onset of nursing with watery diarrhea, meteorism and malnutrition. Previous studies could show that the microvilli reveal a normal shape, but the analysis of enzymatic activities from small intestinal biopsies indicated very low levels of lactase [61,62]. Life-threatening dehydration and electrolyte loss of newborns requires very rapid treatment. The treatment strategy for those patients is the removal of lactose from the diet and the application of milk substitutes. The severeness of this disease occurs because the symptoms are gastrointestinal problems, which might also arise in the case of other diseases. A recent study revealed that severe osmotic diarrhea due to CLD is elicited by severe mutations in the LPH gene that occur in either a compound heterozygous or homozygous pattern of inheritance [87]. Until now, no genetic test has been available to analyze if the parents of an unborn infant might be carriers of a pathogenic mutation in the gene of LPH.

## 6. Secondary Lactose Intolerance Caused by Infections

The secondary LI occurs when the small intestine decreases lactase production after an illness, injury or surgery. Among the diseases associated with secondary LI is celiac disease and Crohn’s disease, but also, infections with various microbes, such as viruses, bacteria and parasites, have been described. In the case of intestinal parasites, Giardia infections were associated with a higher proportion of lactose intolerance in patients from Gabon, Central Africa, analyzed by hydrogen breast tests [89]. In 1991, children with vertically-transmitted human immunodeficiency virus (HIV) infection were evaluated for carbohydrate malabsorption using lactose hydrogen breath tests and d-xylose absorption studies and identified 61% of children with carbohydrate malabsorption. Based on these finding, the authors hypothesized that HIV may be directly involved in the development of lactose malabsorption. Interestingly, a specific interaction of LPH and rotavirus protein NSP4 has been described, which is responsible for the rotavirus-mediated secondary LI [90]. Using cultured human intestinal fully-differentiated enterocyte-like Caco-2 cells, the authors showed that lactase enzymatic activity at the brush border membrane (BBM) is significantly decreased in rhesus monkey rotavirus (RRV)-infected cells. This decreased enzyme activity was not associated with Ca^2+^- and cAMP-dependent signaling events induced by the virus, and furthermore, LPH biosynthesis, stability and protein expression at the BBM was not affected. Instead, the kinetic of lactase enzymatic activity present at the BBM was modified as a result of an inhibitory action of the secreted non-structural rotavirus protein NSP4. NSP4 has pleiotropic functions in viral morphogenesis, as well as pathogenesis [91]. As an example, NSP4 has been shown to alter the F-actin network through the actin-remodeling protein cofilin [92]. Besides viruses and parasites, some bacteria have also been shown to modulate intestinal functions by secreting toxins that lead to the disruption of intestinal epithelial barrier function and subsequent loss of carbohydrate metabolism as, for example, *Clostridium difficile* or [93] *Staphylococcus aureus* toxins [94]. Thus, opportunistic bacteria that use intestine as a reservoir, like methicillin-resistant *Staphylococcus aureus*, may lead to secondary LI-mediated diarrhea during colonization of the host [95,96].

In summary, microbes may use a large variety of sophisticated mechanisms to induce structural and functional lesions at the BBM of human intestinal cells and, thereby, lead to secondary LI. Studying the mechanisms of pathogen-induced damage of epithelial cells might help to develop protective strategies against infection-associated diarrhea.

## 7. Association of Lactase Persistence and Dairy Consumption with the Incidence of Some Common Cancers

With a concentration of about 5% (w/v), lactose is a major ingredient of the milk of cows, sheep and goats [97]. Lactose intolerance appears when lactase activity in the small intestine is insufficient or absent [84,98,99]. The transit of the mal-digested and unabsorbed lactose to the colon is followed by extra bacterial fermentation and production of gas and short chain fatty acids [100,101,102]. Besides irritable influence, these compounds can highly increase the luminal osmolality and interfere with water absorption in the colon, and therefore, cause substantial watery diarrhea. Hypothetically, 12 g of lactose (from about 250 mL of milk) can hold 800 mL of water after processing in the colon [97].

Almost two third of the world population are lactose intolerant because of ATH with a restricted proportion of milk and dairy products in their diet. Besides lactose, dairy foods are nutritious resources of lipids, protein, vitamins and minerals, particularly calcium [103]. In different physiological abnormalities, where dairy foods exert a protective or adverse effect, a comparison between LI and normal subjects has provided an interesting body of evidence for the assessment of the bioactive function of different dairy ingredients in the establishment of health and disease conditions. Colorectal, ovarian and prostate cancers are the most common cancer types in which a protective or adverse effect of milk and dairy foods has been investigated and discussed for a long time [104,105,106,107].

### 7.1. Colorectal Cancer

Colorectal cancer is the fourth most commonly diagnosed and the second cause of fatal cancer in the United States [104]. Diet has a direct relationship with incidence and/or progression of colorectal cancer [108,109]. Consumption of milk, but not cheese that contains saturated fat has been shown to reduce the risk of colorectal cancer [110,111]. A similar protective function has been identified for a calcium- and vitamin D-supplemented diet, attributing the anti-cancer effect of milk to these two biologically-active components [112]. In the intestinal epithelial cells, calcium and vitamin D are shown to regulate cell growth and to promote cell differentiation via stimulation of calcium-sensing receptors [106,113]. Furthermore, in the intestinal lumen, calcium can bind and complex fatty acids, as well as secondary bile acids, and thereby, reduce their cytotoxicity, as well as tumorigenic exposure to the epithelium [114].

In line with these findings, studies in Hungarian and Finnish populations have shown an increased risk for colorectal cancer among LNP subjects [115,116]. However, other studies on Italian, British and Spanish populations do not support such an association [115,117,118]. Based on a meta-analysis of different populations, Szilagyi *et al*. have concluded that the protective effect of dairy food against colorectal cancer is detectable in populations with high or low LNP frequencies, but not in those with significant mixed LNP/LP composition [119,120]. 

Vitamin D can increase calcium absorption in the intestine [121,122]. Therefore, besides life style and nutritional habits, attitudes and exposure to sunshine should also be considered when the effects of dairy food on colorectal cancer are discussed [123]. Interestingly, LNP has been found to have a direct association with sunshine [124,125]. This might be a suggestive evidence for higher LP frequency in higher attitudes as an evolutionary adaptation in order to uptake more of the required vitamin D through dietary sources, especially dairy foods.

### 7.2. Ovarian and Prostate Cancers

Besides the alimentary tract and its associated disorders, such as inflammatory bowel syndrome and colorectal cancer, which are directly exposed to and influenced by the components of milk and dairy foods [126], the pathogenesis of some other parts or organs of the body is also thought to be linked with certain biologically-active ingredients in dairy foods [127]. Thus, the different levels of milk consumption between LP and LNP populations have provided a reasonable model to validate the consistency of the hypotheses regarding beneficial or adverse influences of dairy foods in such conditions. However, conflicting results are available in the literature.

On the one hand, lactose and female sex-related hormones, such as estrogens, in cow’s milk are suspected to play a role in the incidence of ovarian cancer in female or prostate cancer in male subjects [110,128]. The hydrolysis of lactose in the intestine by LP individuals results in the liberation and absorption of glucose and galactose. An excess amount of galactose, especially in the imperfect metabolized form of galactose-1-phosphate, is thought to exert a toxic effect on germ cells, particularly on female ovaries [110,129,130]. Trauma to the surface of the ovary and stimulation of the gonadotropin hormones are two suggested mechanisms for the adverse effect of galactose in the pathogenesis of ovarian cancer [131,132].

In a study of 301 subjects with invasive epithelial ovarian cancer, individuals that belong to the highest category of lactose consumption showed a two-fold elevated risk for serous subtypes of ovarian cancer in comparison to individuals that belong to the lowest category. In this study, each 11 g increase in the lactose intake was found to be associated with 20% higher risk for ovarian cancer [133]. Similar studies by Cramer [134] and Meloni *et al*. [130] have also supported the role of lactose absorption and galactose toxicity in the incidence of ovarian cancer with a higher prevalence for LP women.

On the other side, several publications are found that do not support a correlation of milk consumption with ovarian cancer. One such study is on 327 Finnish, 303 Polish and 152 Swedish subjects genetically determined as LNP *versus* relevant controls, which did not confirm any role for LP and higher milk intake in the etiology of ovarian cancer [105]. Similarly, another study on 108 Caucasian women from western Washington diagnosed with stage I ovarian cancer in comparison to matched controls did not support any correlation between lactose consumption and ovarian cancer [129].

Interestingly, in a cohort study by Koralek *et al*., among 31,925 subjects, a higher consumption of total dairy food was significantly associated with a decreased risk for ovarian cancer [135]. The authors demonstrated that calcium intake reduces the risk for ovarian cancer, similar to what was previously discussed for colorectal cancer. In summary, the evidence for the association of milk consumption and increased risk for prostate cancer is multifactorial and controversial. Current studies do not demonstrate a direct link between LP/LNP genotype and the incidence of prostate cancer; however, some studies have suggested an association between the consumption of dairy products and the development of prostate cancer [136,137]. The investigation of fifty-five prostate cancer subjects by Agarwal *et al*. has determined a lower incidence of LI in these patients in comparison to the general population [138], which is in line with the hypothesis that higher milk intake is a risk factor for prostate cancer [107,139,140]. However, the adverse effect of milk in prostate cancer is considered to be minor and rather suggestive [110,111].

Calcium, insulin-like growth factors, saturated fat and cow’s female hormones are dairy components, and their putative role in prostate cancer is under discussion [141,142]. The estrogens of cow’s milk are proposed to influence the growth of estrogen-sensitive cells in the human body and, thereby, to modulate prostate and ovarian cancers [143,144]. However, it is noteworthy to indicate that in several studies, the levels of biologically-active estrogens in the commercially available milk products have been defined to be far below an effective range to exert such a detrimental effect on consumers [145,146,147,148,149,150,151].

## 8. Concluding Remarks and Outlook

In the intestinal lumen, lactose from milk and dairy foods is hydrolyzed by LPH to glucose and galactose, which are subsequently absorbed. LPH is a membrane-bound glycoprotein of the intestinal epithelial cells. Insufficient levels of lactase activity in the intestine may result in the occurrence of gastrointestinal symptoms upon consumption of milk and dairy products, known as LI. The most common type of LI affecting more than two-thirds of the world population is the ATH or lactase LNP phenotype. This phenotype is caused by reduced activity of the lactase gene promoter after childhood. In LP individuals, different SNPs at 13–14 kbp upstream of the lactase gene have been identified that regulate chromatin changes to keep the lactase expression persistent during adulthood. Other types of LI are induced by inherited mutations in the coding region of LPH, known as alactasia or secondary LI, as a result of the pathogenic defects of the intestinal tissue, as caused by parasitic, bacterial or viral infections.

Besides lactose, milk and dairy foods are rich in other biologically-active compound, which are thought to exert protective or adverse effects on the incidence of colorectal ovarian or prostate cancers. Evidence from comparative studies on LP and LNP cases are more supportive for a protective or at least non-adverse function of milk and dairy food against colorectal and ovarian cancers. The adverse effects of dairy products on prostate cancer are more suggestive than decisive and are found to be mostly linked to the consumption of low fat milk [136]. Furthermore, probiotics in the non-fermented and fermented milk products are shown to balance the gut microbiota and, thereby, exert an immunomodulatory function, which leads to improved gastrointestinal physiology and helps recovery from many gut-associated disorders, including gastro-enteritis, inflammatory bowel disease and constipation [126,152,153,154].

Therefore, milk and dairy foods are not recommended to be ignored in the daily diet. To overcome lactose intolerance syndromes, LNP individuals can follow strategies, such as reducing the amount and increasing the frequency of dairy consumption, as well as using fermented dairy products, which contain active microbial lactase and have a slower bowel transit [100,155].

There is emerging evidence that attributes the occurrence of IBS to the marginal levels of activity of intestinal disaccharidases [156,157]. The contribution of heterozygote mutations in the LCT gene or the LNP phenotype to the appearance of IBS symptoms is an important topic that needs to be investigated in the future. Lower dairy consumption in LNP individuals is associated with a modified gut microbiota [158]. In these cases, the role of altered gut microbiota in the incidence of different intestinal disorders, including IBS and IBD, is an open interesting question, which needs to be further studied.
